# Disilver(I) tricobalt(II) hydrogenphos­phate bis­(phosphate), Ag_2_Co_3_(HPO_4_)(PO_4_)_2_

**DOI:** 10.1107/S1600536811022598

**Published:** 2011-06-18

**Authors:** Abderrazzak Assani, Lahcen El Ammari, Mohammed Zriouil, Mohamed Saadi

**Affiliations:** a Laboratoire de Chimie du Solide Appliquée, Faculté des Sciences, Université Mohammed V-Agdal, Avenue Ibn Battouta, BP 1014, Rabat, Morocco

## Abstract

Ag_2_Co_3_(HPO_4_)(PO_4_)_2_ contains CoO_6_ octa­hedra and phosphate groups linked to form a three-dimensional network defining tunnels parallel to the *a* axis that are occupied by Ag^+^ ions.

## Related literature

Compounds prepared hydro­thermally in the Ag_2_O–*M*O–P_2_O_5_ (*M* = divalent cation) system include AgMg_3_(PO_4_)(HPO_4_)_2_ (Assani *et al.*, 2011*a* ▶), AgMn_3_(PO_4_)(HPO_4_)_2_ (Leroux *et al.*, 1995 ▶), AgCo_3_(PO_4_)(HPO_4_)_2_ (Guesmi & Driss, 2002 ▶), AgNi_3_(PO_4_)(HPO_4_)_2_ (Ben Smail & Jouini, 2002 ▶), Ag_2_Ni_3_(HPO_4_)(PO_4_)_2_ (Assani *et al.*, 2011*b* ▶) and γ-AgZnPO_4_ (Assani *et al.*, 2010 ▶).

## Experimental

### 

#### Crystal data

Ag_2_Co_3_(HPO_4_)(PO_4_)_2_
*M* *_r_* = 678.44
Orthorhombic, 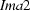

*a* = 12.9814 (4) Å
*b* = 6.5948 (2) Å
*c* = 10.7062 (3) Å
*V* = 916.55 (5) Å^3^
*Z* = 4
Mo *K*α radiation
μ = 10.11 mm^−1^
*T* = 296 K
0.26 × 0.12 × 0.09 mm

#### Data collection

Bruker X8 APEX diffractometer
Absorption correction: multi-scan (*MULABS*; Blessing, 1995 ▶) *T*_min_ = 0.365, *T*_max_ = 0.424
3966 measured reflections
1388 independent reflections
1368 reflections with *I* > 2σ(*I*)
*R*_int_ = 0.021

#### Refinement

*R*[*F* ^2^ > 2σ(*F* ^2^)] = 0.028
*wR*(*F* ^2^) = 0.064
*S* = 1.05
1388 reflections
99 parameters
1 restraint
H-atom parameters constrained
Δρ_max_ = 1.81 e Å^−3^
Δρ_min_ = −1.54 e Å^−3^
Absolute structure: Flack (1983 ▶), 653 Friedel pairs
Flack parameter: 0.55 (3)

### 

Data collection: *APEX2* (Bruker, 2005 ▶); cell refinement: *SAINT* (Bruker, 2005 ▶); data reduction: *SAINT*; program(s) used to solve structure: *SHELXS97* (Sheldrick, 2008 ▶); program(s) used to refine structure: *SHELXL97* (Sheldrick, 2008 ▶); molecular graphics: *ORTEP-3 for Windows* (Farrugia, 1997 ▶) and *DIAMOND* (Brandenburg, 2006 ▶); software used to prepare material for publication: *WinGX* (Farrugia, 1999 ▶).

## Supplementary Material

Crystal structure: contains datablock(s) I, global. DOI: 10.1107/S1600536811022598/mg2119sup1.cif

Structure factors: contains datablock(s) I. DOI: 10.1107/S1600536811022598/mg2119Isup2.hkl

Additional supplementary materials:  crystallographic information; 3D view; checkCIF report

## Figures and Tables

**Table 1:** Hydrogen-bond geometry (Å, °)

*D*—H⋯*A*	*D*—H	H⋯*A*	*D*⋯*A*	*D*—H⋯*A*
O4—H4⋯O4^i^	0.86	1.86	2.626 (7)	148

Symmetry code: (i) 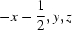.

**Fig. 1.**
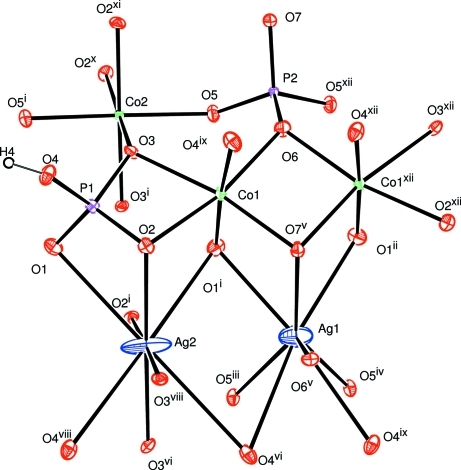
Connectivity of metal-centred coordination polyhedra in Ag2Co3(HPO4)(PO4)2. Displacement ellipsoids are drawn at the 50% probability level. Symmetry codes: (i) -x, -y + 1, z; (ii) x + 1/2, -y+ 1, z; (iii) x, -y + 3/2, z - 1/2; (iv) -x + 1/2, -y + 3/2, z - 1/2; (v) -x + 1/2, -y + 1/2, z - 1/2; (vi) -x, y + 1/2, z - 1/2; (vii) x + 1/2, y + 1/2, z - 1/2; (viii) x, -y + 1/2, z - 1/2; (ix) -x, -y, z; (x) -x, y + 1/2, z + 1/2; (xi) x, -y + 1/2, z + 1/2; (xii) -x + 1/2, y, z.

**Fig. 2.**
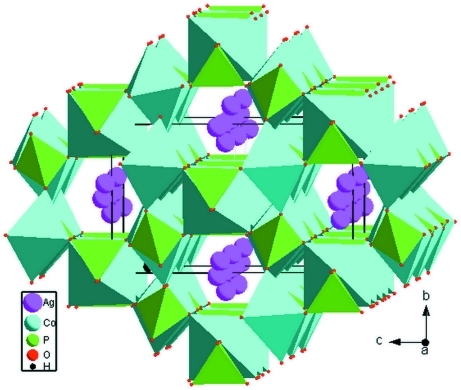
Polyhedral representation of Ag2Co3(HPO4)(PO4)2, showing tunnels running along the a direction at x 1/2 0 and x 0 1/2.

## supplementary crystallographic information

### Comment

Compounds prepared hydrothermally in the Ag_2_O–MO–P_2_O_5_ (M = divalent cation) systems include AgMg_3_(PO_4_)(HPO_4_)_2_ (Assani *et al.*, 2011*a*), AgMn_3_(PO_4_)(HPO_4_)_2_ (Leroux *et al.*, 1995), AgCo_3_(PO_4_)(HPO_4_)_2_ (Guesmi & Driss, 2002), AgNi_3_(PO_4_)(HPO_4_)_2_ (Ben Smail & Jouini, 2002), Ag_2_Ni_3_(HPO_4_)(PO_4_)_2_ (Assani *et al.*, 2011*b*), and γ-AgZnPO_4_ (Assani *et al.*, 2010). Ag_2_Co_3_(HPO_4_)(PO_4_)_2_, isostructural to the Ni analogue, contains CoO_6_ octahedra and PO_4_ and PO_3_(OH) tetrahedra which share corners and edges to form a three-dimensional framework (Fig. 1). Two types of tunnels aligned parallel to the *a*-direction accommodate Ag^+^ cations (Fig. 2).

### Experimental

A mixture of 0.0849 g AgNO_3_, 0.0529 g CoCO_3_.Co(OH)_2_, 10 mL of 85 wt.% H_3_PO_4_, and 10 mL of distilled water was placed in a 23-mL Teflon-lined autoclave, which was heated at 468 K under autogeneous pressure for two days. Pink crystals of the title compound were obtained after the product was filtered, washed with deionized water, and dried in air.

### Refinement

The O-bound H atom was initially located in a difference map and refined with O—H distance restraints of 0.86 (1) in a riding model approximation with *U*_iso_(H) set to 1.2*U*_eq_(O). The highest and deepest hole in the final difference Fourier map are located at 0.70 Å and 0.51 Å, respectively, from Ag1.

### Crystal data

**Table:**

Ag_2_Co_3_(HPO_4_)(PO_4_)_2_	*F*(000) = 1268
*M**_r_* = 678.44	*D*_x_ = 4.917 Mg m^−^^3^
Orthorhombic, *I**m**a*2	Mo *K*α radiation, λ = 0.71073 Å
Hall symbol: I 2 -2a	Cell parameters from 1388 reflections
*a* = 12.9814 (4) Å	θ = 3.1–30.0°
*b* = 6.5948 (2) Å	µ = 10.11 mm^−^^1^
*c* = 10.7062 (3) Å	*T* = 296 K
*V* = 916.55 (5) Å^3^	Prism, pink
*Z* = 4	0.26 × 0.12 × 0.09 mm

### Data collection

**Table:**

Bruker X8 APEX diffractometer	1388 independent reflections
Radiation source: fine-focus sealed tube	1368 reflections with *I* > 2σ(*I*)
graphite	*R*_int_ = 0.021
φ and ω scans	θ_max_ = 30.0°, θ_min_ = 3.1°
Absorption correction: multi-scan (*MULABS*; Blessing, 1995)	*h* = −17→18
*T*_min_ = 0.365, *T*_max_ = 0.424	*k* = −3→9
3966 measured reflections	*l* = −14→15

### Refinement

**Table:**

Refinement on *F*^2^	Secondary atom site location: difference Fourier map
Least-squares matrix: full	Hydrogen site location: inferred from neighbouring sites
*R*[*F*^2^ > 2σ(*F*^2^)] = 0.028	H-atom parameters constrained
*wR*(*F*^2^) = 0.064	*w* = 1/[σ^2^(*F*_o_^2^) + (0.036*P*)^2^ + 2.5641*P*] where *P* = (*F*_o_^2^ + 2*F*_c_^2^)/3
*S* = 1.05	(Δ/σ)_max_ < 0.001
1388 reflections	Δρ_max_ = 1.81 e Å^−^^3^
99 parameters	Δρ_min_ = −1.54 e Å^−^^3^
1 restraint	Absolute structure: Flack (1983), 653 Friedel pairs
Primary atom site location: structure-invariant direct methods	Flack parameter: 0.55 (3)

### Special details

**Table:**

Geometry. All e.s.d.'s (except the e.s.d. in the dihedral angle between two l.s. planes) are estimated using the full covariance matrix. The cell e.s.d.'s are taken into account individually in the estimation of e.s.d.'s in distances, angles and torsion angles; correlations between e.s.d.'s in cell parameters are only used when they are defined by crystal symmetry. An approximate (isotropic) treatment of cell e.s.d.'s is used for estimating e.s.d.'s involving l.s. planes.
Refinement. Refinement of *F*^2^ against all reflections. The weighted *R*-factor *wR* and goodness of fit *S* are based on *F*^2^, conventional *R*-factors *R* are based on *F*, with *F* set to zero for negative *F*^2^. The threshold expression of *F*^2^ > σ(*F*^2^) is used only for calculating *R*-factors(gt) *etc*. and is not relevant to the choice of reflections for refinement. *R*-factors based on *F*^2^ are statistically about twice as large as those based on *F*, and *R*- factors based on all data will be even larger.

### Fractional atomic coordinates and isotropic or equivalent isotropic displacement parameters (Å^2^)

**Table:**

	*x*	*y*	*z*	*U*_iso_*/*U*_eq_	Occ. (<1)
Ag1	0.2500	0.61215 (8)	−0.01381 (7)	0.03097 (16)	
Ag2	0.0000	0.5000	−0.03770 (5)	0.0448 (2)	
Co1	0.13632 (3)	0.24907 (9)	0.20816 (6)	0.00759 (11)	
Co2	0.0000	0.5000	0.45678 (7)	0.00474 (13)	
P1	−0.07308 (7)	0.25700 (17)	0.20656 (12)	0.00728 (17)	
P2	0.2500	0.40742 (18)	0.45614 (14)	0.0051 (2)	
O1	−0.1344 (3)	0.4442 (5)	0.1740 (3)	0.0117 (6)	
O2	0.0039 (3)	0.2072 (5)	0.1002 (3)	0.0084 (6)	
O3	0.0017 (3)	0.2766 (5)	0.3204 (3)	0.0078 (6)	
O4	−0.1489 (3)	0.0787 (5)	0.2349 (3)	0.0116 (7)	
O5	0.15460 (18)	0.5409 (4)	0.4551 (3)	0.0102 (5)	
O6	0.2500	0.2616 (7)	0.3410 (4)	0.0109 (11)	
O7	0.2500	0.2663 (7)	0.5736 (4)	0.0083 (10)	
H4	−0.2103	0.0633	0.2635	0.010*	0.50

### Atomic displacement parameters (Å^2^)

**Table:**

	*U*^11^	*U*^22^	*U*^33^	*U*^12^	*U*^13^	*U*^23^
Ag1	0.0498 (3)	0.0187 (2)	0.0244 (3)	0.000	0.000	0.0049 (2)
Ag2	0.1126 (6)	0.0094 (2)	0.0124 (3)	−0.0022 (2)	0.000	0.000
Co1	0.00546 (19)	0.0103 (2)	0.0070 (2)	0.0005 (2)	0.0001 (2)	−0.00120 (18)
Co2	0.0051 (2)	0.0051 (3)	0.0040 (3)	0.00074 (19)	0.000	0.000
P1	0.0069 (3)	0.0078 (4)	0.0071 (4)	0.0000 (4)	−0.0003 (5)	0.0005 (4)
P2	0.0043 (5)	0.0066 (5)	0.0044 (6)	0.000	0.000	−0.0005 (5)
O1	0.0133 (15)	0.0094 (14)	0.0124 (14)	0.0018 (11)	−0.0027 (10)	0.0002 (11)
O2	0.0096 (17)	0.0072 (12)	0.0083 (14)	−0.0004 (13)	−0.0014 (11)	−0.0031 (13)
O3	0.0080 (17)	0.0091 (14)	0.0063 (13)	0.0030 (12)	−0.0020 (10)	−0.0015 (12)
O4	0.0107 (17)	0.0083 (15)	0.0159 (18)	−0.0018 (11)	0.0039 (10)	0.0001 (10)
O5	0.0067 (9)	0.0115 (10)	0.0123 (13)	0.0014 (9)	0.0003 (12)	0.0000 (12)
O6	0.011 (3)	0.014 (2)	0.008 (2)	0.000	0.000	−0.0033 (15)
O7	0.007 (3)	0.010 (2)	0.0083 (19)	0.000	0.000	0.0021 (15)

### Geometric parameters (Å, °)

**Table:**

Ag1—O1^i^	2.537 (3)	Co2—O2^xi^	2.056 (3)
Ag1—O1^ii^	2.537 (3)	Co2—O3^i^	2.074 (3)
Ag1—O5^iii^	2.623 (3)	Co2—O3	2.074 (3)
Ag1—O5^iv^	2.623 (3)	P1—O1	1.510 (3)
Ag1—O7^v^	2.666 (4)	P1—O2	1.550 (4)
Ag1—O6^v^	2.914 (5)	P1—O4	1.563 (3)
Ag1—O4^vi^	3.001 (3)	P1—O3	1.563 (4)
Ag1—O4^vii^	3.001 (3)	P2—O5	1.519 (3)
Ag1—Ag2	3.33837 (16)	P2—O5^xii^	1.520 (3)
Ag2—O3^viii^	2.374 (3)	P2—O6	1.563 (5)
Ag2—O3^vi^	2.374 (3)	P2—O7	1.564 (5)
Ag2—O2	2.431 (4)	O1—Co1^i^	2.055 (3)
Ag2—O2^i^	2.431 (4)	O1—O4	2.504 (4)
Ag2—O1	2.884 (3)	O1—O2	2.509 (5)
Ag2—O1^i^	2.884 (3)	O1—Ag1^i^	2.536 (3)
Ag2—O4^viii^	3.151 (3)	O2—Co2^xiii^	2.056 (3)
Ag2—O4^vi^	3.151 (3)	O3—Ag2^xiv^	2.374 (3)
Ag2—Ag1^i^	3.33837 (16)	O4—Ag1^xv^	3.001 (3)
Co1—O6	2.051 (3)	O4—Ag2^xiv^	3.151 (3)
Co1—O1^i^	2.055 (3)	O4—H4	0.8598
Co1—O7^v^	2.065 (3)	O5—Ag1^xvi^	2.623 (3)
Co1—O2	2.090 (3)	O6—Co1^xii^	2.051 (3)
Co1—O3	2.128 (4)	O6—Ag1^xvii^	2.914 (5)
Co1—O4^ix^	2.187 (3)	O7—Co1^xi^	2.065 (3)
Co2—O5^i^	2.025 (2)	O7—Co1^xvii^	2.065 (3)
Co2—O5	2.025 (2)	O7—Ag1^xvii^	2.666 (4)
Co2—O2^x^	2.056 (3)		
			
O1^i^—Ag1—O1^ii^	72.52 (15)	O2—Ag2—O4^vi^	125.96 (10)
O1^i^—Ag1—O5^iii^	87.09 (10)	O2^i^—Ag2—O4^vi^	110.56 (10)
O1^ii^—Ag1—O5^iii^	120.52 (10)	O1—Ag2—O4^vi^	177.68 (9)
O1^i^—Ag1—O5^iv^	120.52 (10)	O1^i^—Ag2—O4^vi^	102.42 (8)
O1^ii^—Ag1—O5^iv^	87.09 (10)	O4^viii^—Ag2—O4^vi^	78.84 (11)
O5^iii^—Ag1—O5^iv^	56.35 (11)	Ag1^i^—Ag2—Ag1	171.21 (3)
O1^i^—Ag1—O7^v^	65.44 (10)	O6—Co1—O1^i^	95.28 (16)
O1^ii^—Ag1—O7^v^	65.44 (10)	O6—Co1—O7^v^	88.38 (11)
O5^iii^—Ag1—O7^v^	149.47 (7)	O1^i^—Co1—O7^v^	86.15 (16)
O5^iv^—Ag1—O7^v^	149.47 (7)	O6—Co1—O2	168.69 (14)
O1^i^—Ag1—O6^v^	107.39 (11)	O1^i^—Co1—O2	91.26 (14)
O1^ii^—Ag1—O6^v^	107.39 (11)	O7^v^—Co1—O2	101.28 (12)
O5^iii^—Ag1—O6^v^	132.08 (10)	O6—Co1—O3	101.28 (13)
O5^iv^—Ag1—O6^v^	132.08 (10)	O1^i^—Co1—O3	90.38 (13)
O7^v^—Ag1—O6^v^	52.78 (11)	O7^v^—Co1—O3	170.01 (13)
O1^i^—Ag1—O4^vi^	116.18 (10)	O2—Co1—O3	69.40 (10)
O1^ii^—Ag1—O4^vi^	163.45 (9)	O6—Co1—O4^ix^	84.00 (15)
O5^iii^—Ag1—O4^vi^	75.15 (9)	O1^i^—Co1—O4^ix^	175.52 (12)
O5^iv^—Ag1—O4^vi^	99.02 (9)	O7^v^—Co1—O4^ix^	89.40 (15)
O7^v^—Ag1—O4^vi^	104.24 (11)	O2—Co1—O4^ix^	90.18 (13)
O6^v^—Ag1—O4^vi^	57.31 (10)	O3—Co1—O4^ix^	94.10 (13)
O1^i^—Ag1—O4^vii^	163.45 (9)	O5^i^—Co2—O5	178.97 (18)
O1^ii^—Ag1—O4^vii^	116.18 (10)	O5^i^—Co2—O2^x^	94.06 (13)
O5^iii^—Ag1—O4^vii^	99.02 (9)	O5—Co2—O2^x^	86.71 (13)
O5^iv^—Ag1—O4^vii^	75.15 (9)	O5^i^—Co2—O2^xi^	86.71 (13)
O7^v^—Ag1—O4^vii^	104.24 (11)	O5—Co2—O2^xi^	94.06 (13)
O6^v^—Ag1—O4^vii^	57.31 (10)	O2^x^—Co2—O2^xi^	83.4 (2)
O4^vi^—Ag1—O4^vii^	51.87 (13)	O5^i^—Co2—O3^i^	94.45 (13)
O3^viii^—Ag2—O3^vi^	100.42 (17)	O5—Co2—O3^i^	84.82 (13)
O3^viii^—Ag2—O2	77.19 (9)	O2^x^—Co2—O3^i^	93.07 (11)
O3^vi^—Ag2—O2	177.52 (14)	O2^xi^—Co2—O3^i^	176.32 (16)
O3^viii^—Ag2—O2^i^	177.52 (14)	O5^i^—Co2—O3	84.82 (13)
O3^vi^—Ag2—O2^i^	77.19 (9)	O5—Co2—O3	94.45 (13)
O2—Ag2—O2^i^	105.21 (15)	O2^x^—Co2—O3	176.32 (16)
O3^viii^—Ag2—O1	114.25 (11)	O2^xi^—Co2—O3	93.07 (11)
O3^vi^—Ag2—O1	126.53 (11)	O3^i^—Co2—O3	90.51 (18)
O2—Ag2—O1	55.53 (11)	O1—P1—O2	110.1 (2)
O2^i^—Ag2—O1	67.13 (10)	O1—P1—O4	109.14 (18)
O3^viii^—Ag2—O1^i^	126.53 (11)	O2—P1—O4	112.90 (19)
O3^vi^—Ag2—O1^i^	114.25 (11)	O1—P1—O3	116.08 (18)
O2—Ag2—O1^i^	67.13 (10)	O2—P1—O3	100.93 (14)
O2^i^—Ag2—O1^i^	55.53 (11)	O4—P1—O3	107.57 (19)
O1—Ag2—O1^i^	76.38 (13)	O1—P1—Co1	122.99 (14)
O3^viii^—Ag2—O4^viii^	52.04 (11)	O5—P2—O5^xii^	109.2 (2)
O3^vi^—Ag2—O4^viii^	68.06 (10)	O5—P2—O6	110.52 (15)
O2—Ag2—O4^viii^	110.56 (10)	O5^xii^—P2—O6	110.52 (15)
O2^i^—Ag2—O4^viii^	125.96 (10)	O5—P2—O7	110.52 (15)
O1—Ag2—O4^viii^	102.42 (8)	O5^xii^—P2—O7	110.53 (15)
O1^i^—Ag2—O4^viii^	177.68 (9)	O6—P2—O7	105.5 (2)
O3^viii^—Ag2—O4^vi^	68.06 (10)	P1—O4—H4	137.9
O3^vi^—Ag2—O4^vi^	52.04 (11)		

Symmetry codes: (i) −*x*, −*y*+1, *z*; (ii) *x*+1/2, −*y*+1, *z*; (iii) *x*, −*y*+3/2, *z*−1/2; (iv) −*x*+1/2, −*y*+3/2, *z*−1/2; (v) −*x*+1/2, −*y*+1/2, *z*−1/2; (vi) −*x*, *y*+1/2, *z*−1/2; (vii) *x*+1/2, *y*+1/2, *z*−1/2; (viii) *x*, −*y*+1/2, *z*−1/2; (ix) −*x*, −*y*, *z*; (x) −*x*, *y*+1/2, *z*+1/2; (xi) *x*, −*y*+1/2, *z*+1/2; (xii) −*x*+1/2, *y*, *z*; (xiii) −*x*, *y*−1/2, *z*−1/2; (xiv) −*x*, *y*−1/2, *z*+1/2; (xv) *x*−1/2, *y*−1/2, *z*+1/2; (xvi) −*x*+1/2, −*y*+3/2, *z*+1/2; (xvii) −*x*+1/2, −*y*+1/2, *z*+1/2.

### Hydrogen-bond geometry (Å, °)

**Table:**

*D*—H···*A*	*D*—H	H···*A*	*D*···*A*	*D*—H···*A*
O4—H4···O4^xviii^	0.86	1.86	2.626 (7)	148.

Symmetry codes: (xviii) −*x*−1/2, *y*, *z*.
